# Polyol-Assisted Synthesis of Ni/Cu/Ag Trimetallic Nanoparticles for Nonlinear Optical Applications

**DOI:** 10.1021/acsomega.4c03143

**Published:** 2024-11-15

**Authors:** Shilpa Molakkalu Padre, Shivakumar Jagadish Shetty, Saideep Shirish Bhat, Desmond Gracian Rebello, Srivathsava Surabhi, Shreya Rao, Neelamma Basayya Gummagol, Maqsood Rafique Waikar, Rajendra Girjappa Sonkawade, Gurumurthy Sangam Chandrasekhar

**Affiliations:** † Nano and Functional Materials Lab (NFML), Department of Physics, Manipal Institute of Technology, Manipal Academy of Higher Education, Manipal 576104, Karnataka, India; ‡ Laboratorio de Nanocompuestos, Departamento de Ingeniería de Materiales (DIMAT), Facultad de Ingeniería (FI), Universidad de Concepción (UdeC), Concepción 4030000, Chile; § Laboratorio de Nanociencias y Nanotecnología, Facultad de Ciencias Físico Matemáticas (FCFM), Universidad Autónoma de Nuevo León (UANL), San Nicolás de los Garza, Nuevo León 66451, Mexico; ∥ Department of Physics, School of Advanced Sciences, KLE Technological University, Vidhyanagar, Hubballi 580031, Karnataka, India; ⊥ Radiation and Materials Research Laboratory, Department of Physics, Shivaji University, Kolhapur 416004, Maharashtra, India

## Abstract

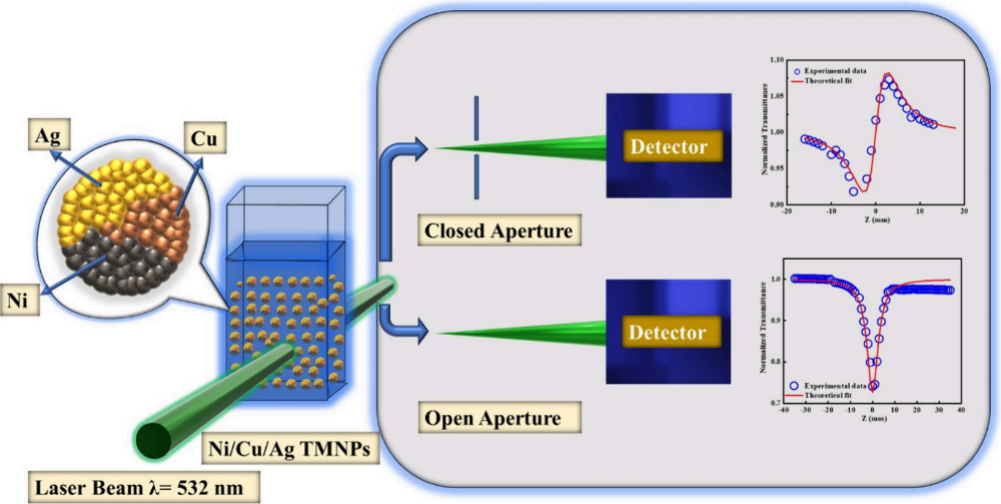

Trimetallic nanoparticles (TMNPs) have opened a broad spectrum of applications with a new class of materialistic combinations in several fields from electronics to medicinal and environmental applications. In this work, we report the synthesis and characterization of Ni/Cu/Ag TMNPs using the polyol method and their nonlinear optical (NLO) studies. A broad surface plasmon resonance (SPR) peak at 443 nm evidences the formation of the Ni/Cu/Ag TMNPs with a peak shift compared to their mono- or bimetallic counterparts. The NLO studies showed promising results, indicating that Ni/Cu/Ag TMNPs have potential applications in optoelectronics. The calculated nonlinear absorption coefficient (β) confirmed thermally induced excited-state absorption by the prepared TMNPs. Closed-aperture z-scan analysis demonstrated a self-focusing effect, indicating a nonlinear refractive index (n_2_). Further, the suitability of Ni/Cu/Ag TMNPs for optical limiting application is assessed.

## 1 Introduction

The evolution of hybrid nanostructures (NSs) in terms of core–shell, alloy, or contact aggregation of metallic materials has transfigured areas like optoelectronics, biosensing, catalysis, biomedical applications, and many more.^1^ Subtle compositional variation will afford unique physicochemical characteristics to engineer their optical, sensing, catalytic, and other properties for relevant applications. Fundamentally, these hybrid NSs encompass mono- and bimetallic nanoparticles (BMNPs),^2−6^ whose structural and morphological aspects are governed by synergistic effects through localized surface plasmon resonance (LSPR). Alloying the NSs manipulates their shape and size as well as steers the plasmon-enhanced catalysis through optical absorption.^7^ Harvesting the LSPR-administered optical near-fields and photothermally generated hot electrons at the nanoscale yielded exceptional results in the building of futuristic applications.

In the last two decades, there has been a driven interest toward ternary metallic NSs by combining three metals to exploit the combinatorial effect owing to their superior LSPR-induced catalyzation of the reactions.^8^ Most of the reported literature deals with the well-known noble metals (Au, Ag) as key components in TMNPs.^9^ PdCuAg,^1^ AgAuPd,^10^ PdPtCo,^11^ and (NiCu)@Ag^12^ are a few others that have been researched for diverse applications. However, integrating noble metals with cost-affordable metals, such as Cu, Ni, Co, Fe, etc., to leverage their exceptional qualities may evidence the exceptional manipulation of optical and catalytic activities. However, these materials possess high oxidative sensitivity and agglomeration tendency, which hinder their utility for practical applications. Hence, coalescing them with noble metals could result in TMNPs with the desired and tunable properties.

TMNPs can be synthesized by various methods. The most commonly employed routes include simultaneous and sequential reduction techniques. For instance, TMNPs like Pd/Ag/Fe and Au@PdPt are typically prepared via simultaneous reduction using hydrazine and ascorbic acid as reducing agents.^13,14^ On the other hand, TMNPs such as carbon-supported Pd/Au/Ni and Ag/Au/Pt are synthesized through sequential reduction reactions, employing sodium borohydride and ascorbic acid as reducing agents, respectively.^15,16^ The coprecipitation method is another well-known method to prepare transition metal-based TMNPs such as Fe-Ni-Ce,^17^ Fe-Ni-Zn, Fe-Co-Zn, Co-Ni-Zn,^18^ and Fe-Co-Cu^19^. Additionally, PdNiAl,^20^ NiFeCo,^21^ and PtCuCo,^22^ are synthesized using an environmentally friendly hydrothermal method. The microwave-assisted preparation route is another eco-friendly method for preparing TMNPs, such as AuPtCu,^23^ AgRuNi,^24^ and AgTiZn.^25^ TMNPs such as Pt/Pd/Fe,^26^ carbon-supported Pd/Co/Au,^27^ and Sn-Ni-Cu^28^ are prepared via the microemulsion method to achieve precise control over size. The polyol method is one of the green and cost-effective methods that use high boiling poly alcohol (polyol) as a solvent and stabilizer that attracted significant attention owing to control over the size, shape, composition, and crystallinity of the synthesized nanomaterials.^29,30^ Many studies have reported the polyol method for synthesizing TMNPs due to its simplicity in preventing agglomeration and oxidation. In addition to that, it provides excellent control over structural tunability. Moreover, it is one of the most feasible routes for preparing transition-metal-based TMNPs.^31−33^ In the current study, the polyol method is employed for the first time to prepare Ni/Cu/Ag TMNPs free of Au and Pt.

Further, the NLO properties of metallic nanomaterials represent a compelling area of research due to their potential applications in advanced photonics and optoelectronics. These properties, resulting from the interaction of intense light fields with the nanomaterials, give rise to phenomena such as nonlinear refraction, two-photon absorption, and thermo-optic effects, making them highly suitable for optical limiting, switching, sensing, and imaging applications.^34^ Among metal NSs, noble metals such as gold and silver have garnered significant research interest due to their broad SPR absorption.^35−38^ However, their limited abundance and high cost limit their widespread application. Therefore, researchers are pursuing novel metallic-based NSs with significant NLO response due to their potential in optical device applications. Composites of plasmonic NSs with other materials^39−42^ as well as bimetallic nanomaterials^43−45^ are the most explored NLO materials for photonic applications. Although many researchers investigated the NLO properties of bimetallic nanomaterials based on noble metals,^46−49^ studies on TMNPs for NLO applications are still not explored.

The present work investigates the viability of fabricated TMNPs via polyol route for NLO applications. To the best of our knowledge, this is the first report on Ni/Cu/Ag-based TMNPs to probe their LSPR-based optical sensitivity while yielding excellent structural and morphological properties. Further, the study of NLO parameters under continuous wave (CW) laser at the wavelength 532 nm has portrayed the aptness of the prepared TMNPs to be employed in optical limiters, which is nowhere discussed in the literature.

## 2 Results and Discussion

### 2.1 Optical and Structural Studies

**Figure 1:**
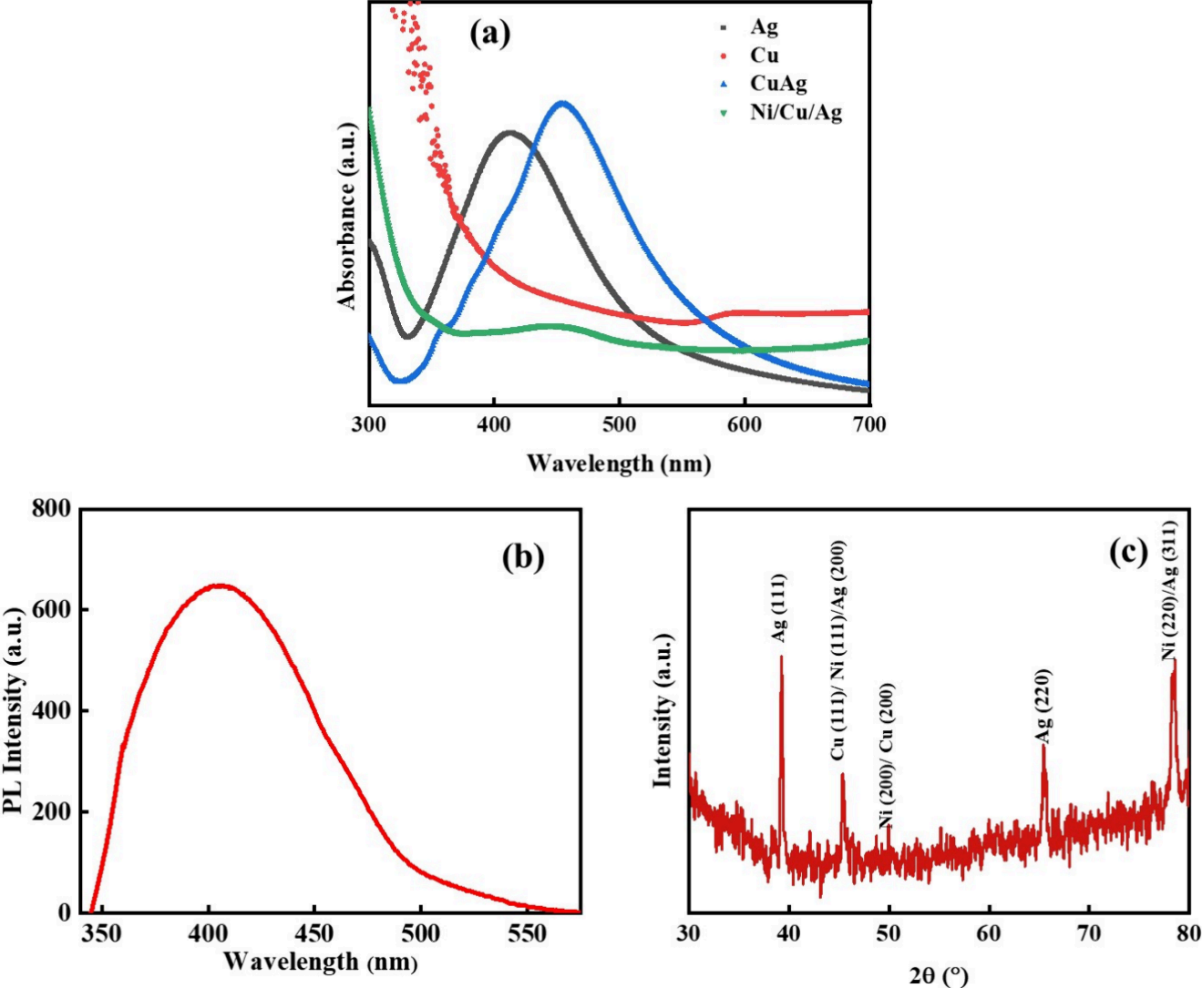
(a) UV–vis absorption spectra of Ag NPs, Cu NPs, CuAg BMNPs, and Ni/Cu/Ag TMNPs. (b) PL spectra and (c) XRD pattern of Ni/Cu/Ag TMNPs.

Figure 1(a) shows that Ag and Cu NPs depict absorption peaks at 412 and 590 nm, respectively. Absorption spectra of the bimetallic CuAg NPs has a broad peak ranging from 400 to 500 nm, and the maximum is at 455 nm. Compared to pure Ag NPs, the SPR peak corresponding to CuAg BMNPs shows a bathochromic shift, and the proximity of this peak to that of Ag NPs is due to the dominance of Ag NPs.^50^ The Ni/Cu/Ag TMNPs depict a peak at 446 nm, slightly red-shifted and broadened compared to Ag NPs. Further, the latter peak has been blue-shifted compared to the SPR peak of CuAg BMNPs and damped substantially, which can be accredited to the damping of the Ni peak by the SPR effect of noble metals.^12,51^ The shift from the SPR peaks of their monometallic or bimetallic counterparts indicates that the combination of nickel, copper, and silver in a single nanoparticle, i.e., in the trimetallic form, results in unique optical properties, which are not observed in their individual or binary forms. The formation of TMNPs likely leads to changes in electron density and plasmonic behavior, reflected in the observed shift in the SPR peak.^52^ Further, the variation of the extinction coefficient (*k*) with respect to wavelength is obtained by using the following equation and plotted in Figure S3(a).
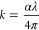
1Here, α and λ are the linear absorption coefficient and wavelength, respectively. It is clear from this figure that the value of *k* decreases with wavelength. As the extinction coefficient measures how strongly a material absorbs or reflects light at a particular wavelength, high values of *k* in the lower wavelength range show that the prepared TMNPs are opaque in this range. At SPR wavelength, the *k* value approaches its maximum^53,54^ (Figure S3(a)). Similarly, the variation of the refractive index with wavelength for the prepared TMNPs in an ethylene glycol medium is plotted in Figure S3(b) using the following equation.^55^
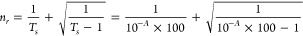
2

Figure 1(b) displays the photoluminescence (PL) spectra of Ni/Cu/Ag TMNPs. Upon excitation at 325 nm, the TMNPs demonstrate an emission peak near 405 nm. This PL peak in the visible range of the trimetallic alloy structure can be attributed to the transition of electrons from the d bands to higher energy states within the Fermi level. Additionally, interactions involving electron–phonon and hole–phonon scattering contribute to energy dissipation, ultimately resulting in the radiative recombination of an electron from the sp band with a hole. The presence of a single emission band further supports the formation of TMNPs; otherwise, a physical mixture of the monometallic counterparts would likely produce distinct peaks.^50,56^

Figure 1(c) depicts the X-ray diffraction (XRD) spectra of the prepared sample. The peaks observed at 39.16° and 65.48° are due to the Ag planes, while the peak at 45.42° is due to the Ag (200), Cu (111), and Ni (111) planes. The peak at 78.33° corresponds to crystal facets of both Ni and Ag^50,57^ as depicted in Figure 1c. Moreover, the dominance of Ag-related peaks in XRD may be attributed to Ag’s higher crystal growth rate, which is in accordance with the literature.^57^ The *d*-spacing values corresponding to the different planes are calculated as follows using Bragg's formula and are tabulated in Table 1.
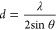
3Here, λ, d, and θ values are the wavelength of the X-ray used, spacing between two consecutive planes, and position of the peak, respectively. The crystallite size of the prepared TMNPs is determined to be 30.25 nm by using the Debye–Scherrer formula,
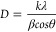
4where k, λ, and β are the Scherrer constant, the X-ray wavelength, and the full width at half-maximum, respectively.^58^

**Table 1:** XRD Parameters of TMNPs.

2θ (°)	β (radian)	D (nm)	d (nm)	ε (× 10^–5^)	δ (×10^14^ m^–2^)	g (%)
39.26	0.21	38	0.22	89.07	6.60	1.06
45.40	0.24	35	0.19	97.82	7.97	1.01
65.49	0.38	24	0.14	141.37	16.64	1.05
78.41	0.42	24	0.12	142.92	17.01	0.90

The microstrain (ε), dislocation density (δ), and distortion parameter (g) are calculated using the following equations^59^ and are tabulated in Table 1.
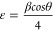
5
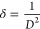
6
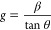
7

### 2.2 Morphological and Elemental Analyses

**Figure 2:**
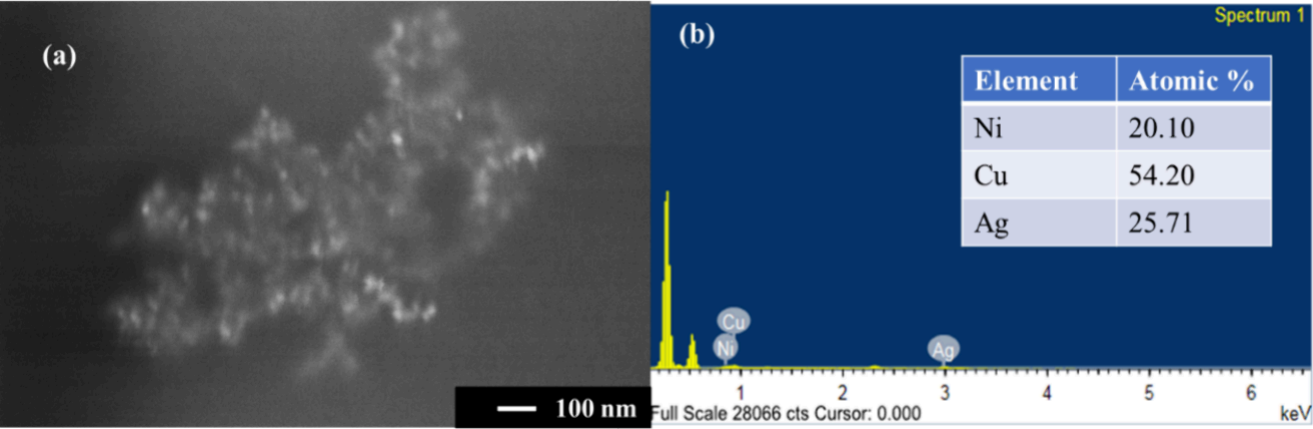
(a) FESEM image of Ni/Cu/Ag TMNPs. (b) Energy-dispersive X-ray spectra of TMNPs.

Figure 2(a) depicts the field emission scanning electron microscopy (FESEM) of Ni/Cu/Ag TMNPs, from which the particles are found to be spherical, with sizes ranging between 35 and 45 nm, which is further validated by transmission electron microscopy (TEM) images. Figure 2(b) depicts the atomic % of the Cu, Ni, and Ag nanoparticles in TMNPs.

**Figure 3:**
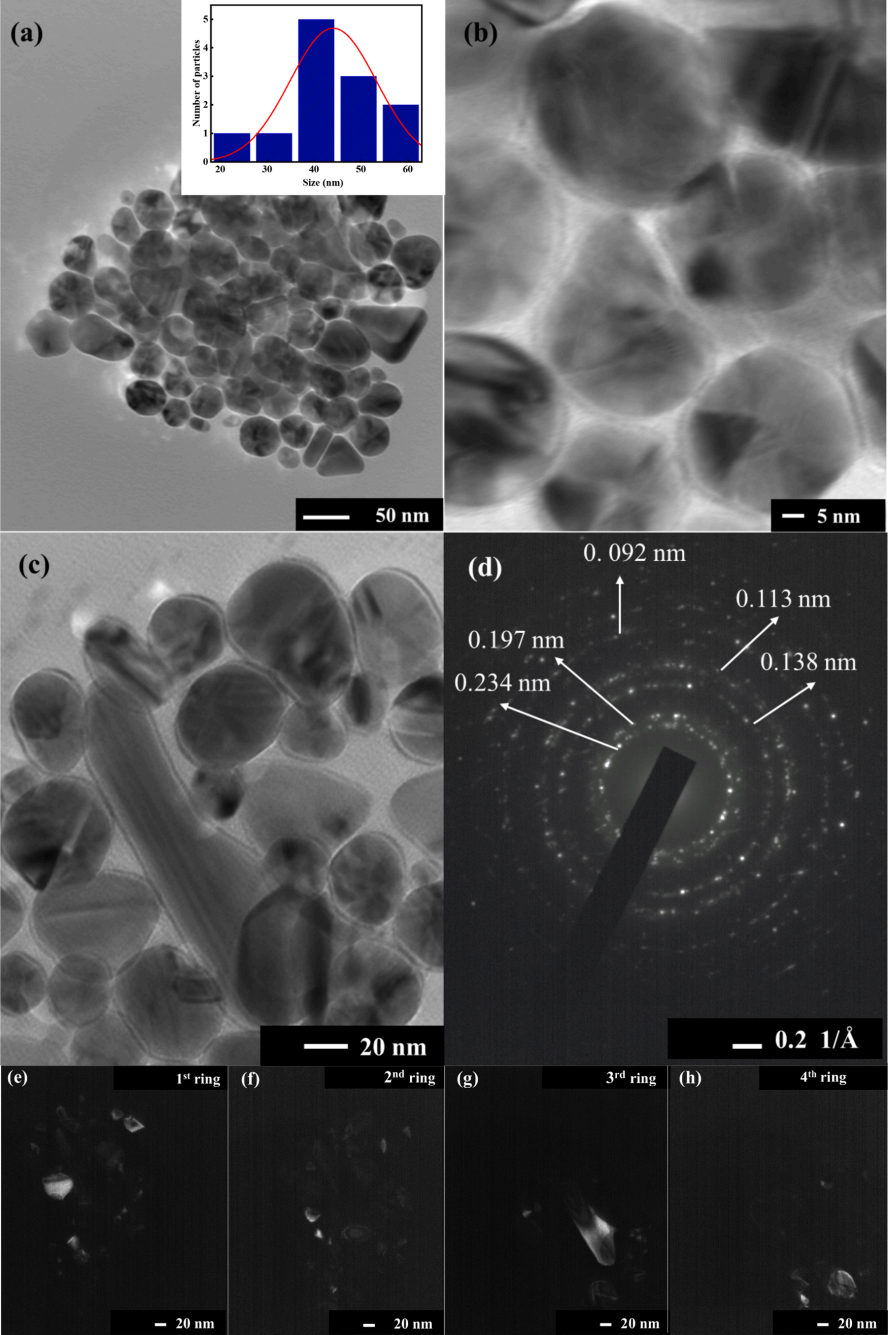
(a–c) TEM images of TMNPs and (d) SAED pattern obtained from Figure 3(c). (e–h) Dark-field images corresponding to the given SAED pattern. Inset: Particle size distribution histogram.

TEM analysis was carried out to get more insight into the morphology. TEM images portray the alloy-like Ni/Cu/Ag TMNPs in Figure 3(a and b). The nonuniform distribution of particles consists of a majority of more or like spherical-shaped TMNPs with an average size of ∼ 44 nm. The thin layer of coating around TMNPs is due to the capping of PVP, which prevents their agglomeration and oxidation, as indicated in Figure 3(c).^60^ The presence of monometallic entities (Ni, Cu, and Ag) can be differentiated by considering the difference in electron density, as specified in Figure 3(b). However, it is difficult to identify the presence of different elements by contrasting the HRTEM image. Hence, the dark-field images are used to distinguish the monometallic counterparts. The dotted rings in the selected area electron diffraction (SAED) pattern obtained from Figure 3(c) depict lattice fringes with crystal facets Ag (111), Cu (111)/ Ni (111)/Ag (200), Ag (220), Ag (222)/Cu (311), and Ag (331)/Cu (400), having *d*-spacing values of 0.234, 0.197, 0.138, 0.113, and 0.092 nm, respectively (Figure 3(d)). The dark-field images corresponding to the aforementioned rings (Figure 3(e–h)) portray the portions corresponding to the Ni, Cu, and Ag elements, thus confirming the formation of a trimetallic alloy-like structure.

**Figure 4:**
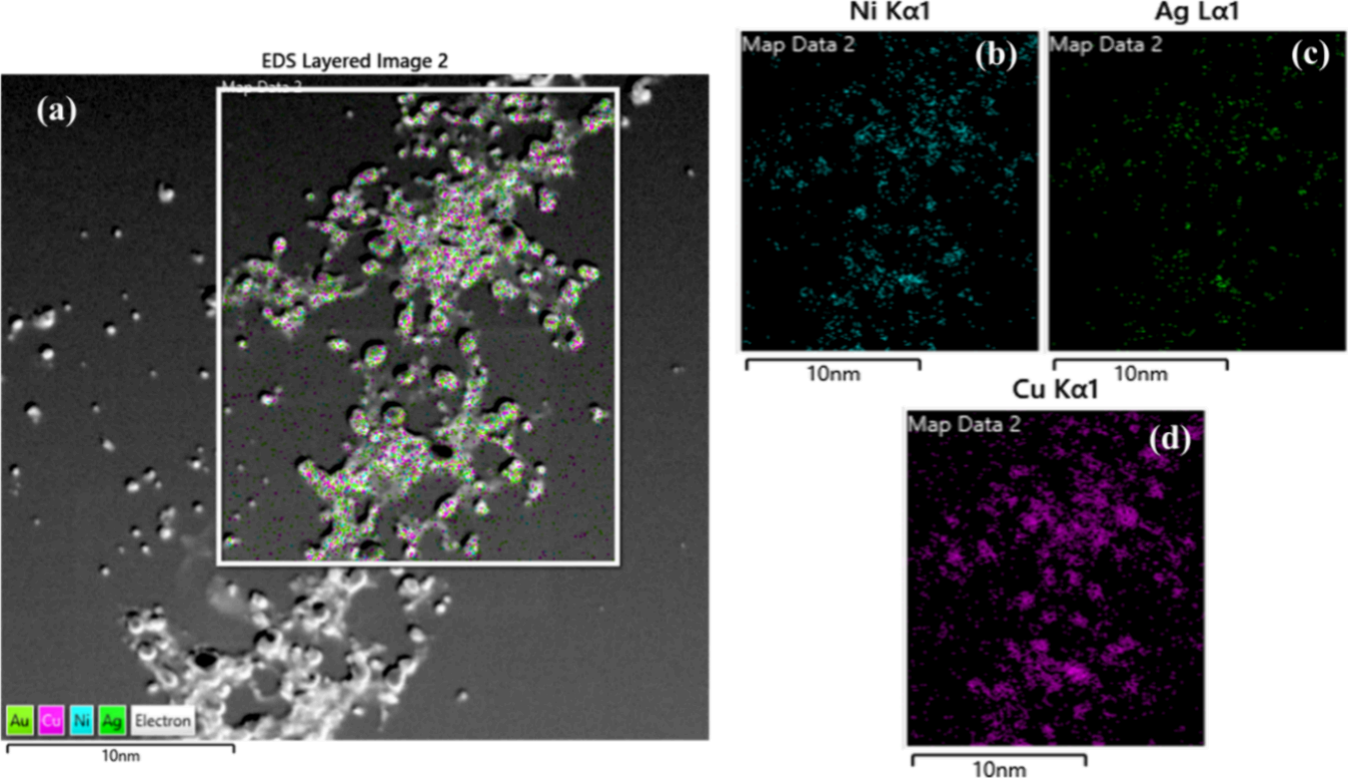
(a–d) Representative false-color EDS elemental maps.

The elemental mapping of the prepared TMNPs is depicted in Figure 4(a–d). It shows the homogeneous distribution of all of the individual elements throughout the NS.

### 2.3 XPS Studies

**Figure 5:**
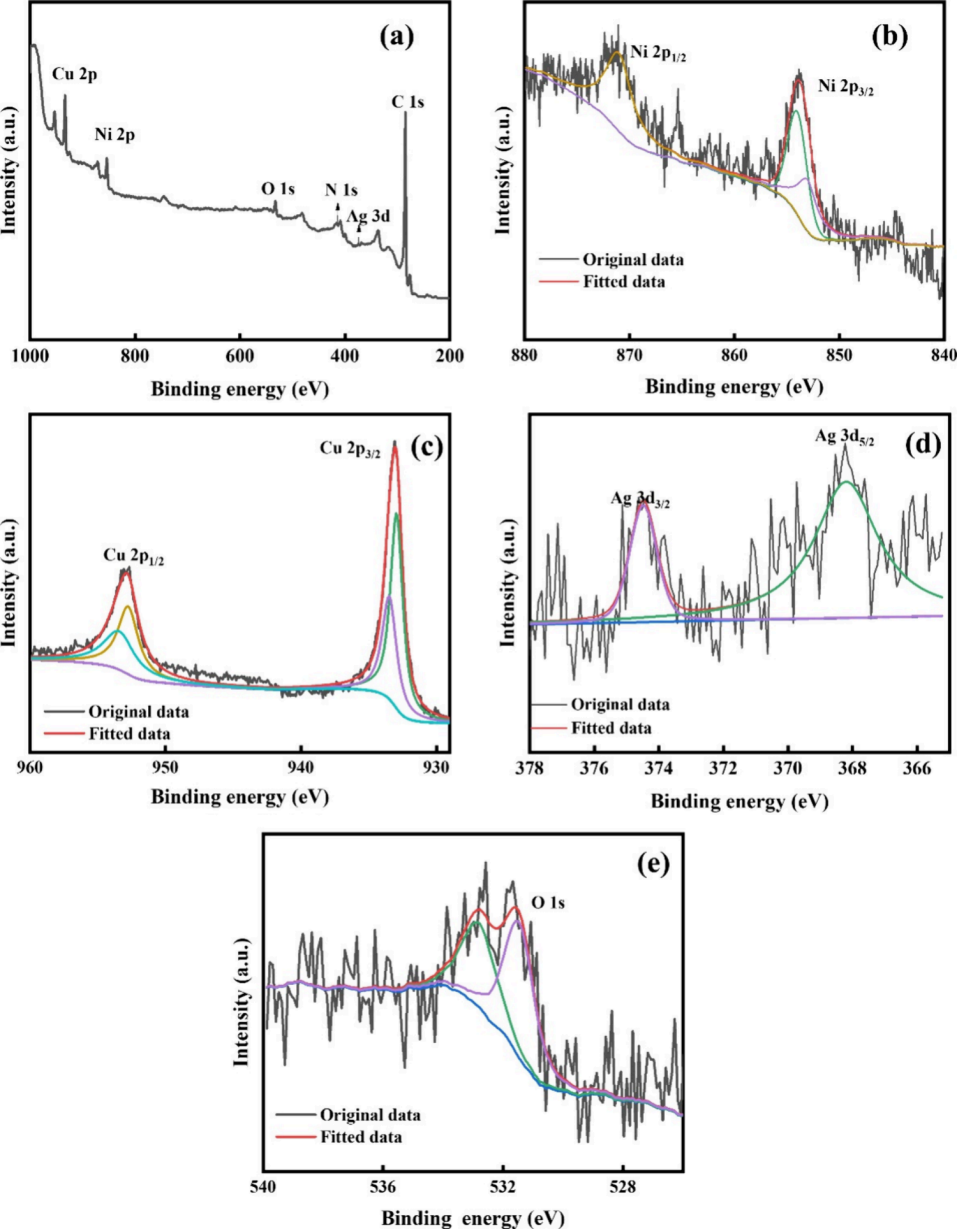
(a) Survey spectra, HR-XPS of (b) Ni 2p, (c) Cu 2p, (d) Ag 3d, and (e) O 1s of TMNPs.

X-ray photoelectron spectroscopy (XPS) analysis was used to confirm the existence of metallic counterparts in the zerovalent form and the possibility of oxidation and interaction with the capping agent. Figure 5(a) indicates the survey spectra and deconvoluted Ni 2p, Cu 2p, Ag 3d, and O 1s spectra of TMNPs. In the Cu 2p spectra, the peaks at 932.92 and 952.84 eV correspond to Cu 2p_3/2_ and Cu 2p_1/2_ of metallic copper. The separation between Cu 2p_1/2_ and Cu 2p_3/2_ is 20.2 eV, which agrees with the established values of Cu (0). Further, the two additional less intense peaks at 933.46 and 953.62 eV are due to the oxidation of Cu. However, the O 1s spectra do not contain any peaks related to higher valent states of either Cu or Ni, clearly indicating the oxidation stability of the prepared TMNPs. The 531.52 and 532.80 eV peaks arise from O_2_ and H_2_O adsorbed on the sample. The Ni 2p core spectra show a slight shifting of Ni peaks, which might be due to the interaction of Ni with PVP (capping agent), which is validated by the presence of a small N 1s peak in the survey spectra.^61^ The Ag 3d peak can be deconvoluted into Ag 3d_3/2_ and Ag 3d_5/2_ with a spin–orbit separation of 6.3 eV. The slightly larger value compared to that of zerovalent Ag can be ascribed to the existence of silver oxidation states.^62^ However, the elements within the depth limit of 4–6 nm are detectable, which might result in a very low intense Ag 3d peak in survey spectra as well as in the fitted graph. Encapsulation of PVP further limits the penetration depth of X-rays.^63^

### 2.4 NLO Studies

When a material is subjected to a strong optical field, such as laser light, its nucleus and electrons experience distortion in their spatial distribution, creating dipoles. However, the induced polarity in these dipoles does not follow the linear relationship with the electric field, resulting in peculiar optical properties, known as NLO properties. As the material returns to its initial state, it emits energy in the form of a radiation with various wavelengths. The polarization induced at higher electric fields is given by the expression^64^
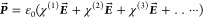
8Here, χ^(1)^ is the linear susceptibility and χ^(2)^ and χ^(3)^ are the second- and third-order nonlinear susceptibilities, respectively.

The present investigation used the polyol method for synthesizing samples, followed by conducting open-aperture (OA) and closed-aperture (CA) Z-scan analyses. Additionally, the optical limiting performance of the prepared samples was evaluated.^65^ The OA mode was employed to determine the nonlinear absorption coefficient, where the transmittance was found to vary with the intensity of the incident light. A material is considered as a saturable absorber (SA) when its transmittance rises with a rise in incident intensity and achieves its maximum value at the focal point.^66^ Conversely, the material is termed as a reverse saturable absorber (RSA) if the transmittance decreases to its lowest value close to the focus point.

**Figure 6:**
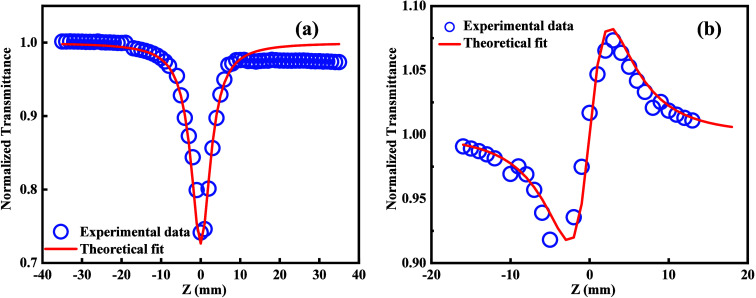
(a) Open-aperture (OA) Z-scan curves of Ni/Cu/Ag TMNPs. (b) Closed-aperture (CA) Z-scan curves of Ni/Cu/Ag TMNPs.

This study found that the transmittance of Ni, Cu, and Ag exhibited a significant decline as the sample position approached the focal point. At the focal point, a sharp dip was observed in the OA curve (Figure 6(a)), indicating the presence of an RSA. In this case, thermal-induced excited-state absorption might be the dominant mechanism responsible for RSA’s nature, where absorption increases due to thermal effects rather than directly from two-photon absorptions.^67,68^ This might arise from the LSPR effect and interband transitions, also reported in previous studies on the NLO properties of BMNPs.^69,70^ The multimetallization of nanoparticles enhances the NLO properties due to the modified electronic structure and d-band perturbation, resulting in increased interband absorption. The following relation gives the normalized transmittance:
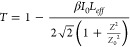
9Using the above equation, one can find the nonlinear absorption coefficient, β.^71^ In this equation, the intensity at the focus is represented by I_0_. At the same time, the effective propagation length is given by L= (1– *e*xp(-α_0_L))/α_*0*_, where α_*0*_ and L correspond to the linear absorption coefficient and the actual path length, respectively. Z denotes the displacement from the focal point, whereas Z_0_ represents the Rayleigh length. For Ni/Cu/Ag, the calculated value of β was 4.12 × 10^–5^ cm W^−1^.

The CA mode determines the nonlinear refractive index (n_2_).
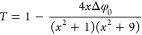
10Here, x = Z/Z_0_, Δφ_0_ is the phase change at the focal point, and the third-order nonlinear refractive index is given by the following expression:
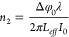
11

The CA Z-scan plot (Figure 6(b)) shows a valley, followed by a peak, a characteristic feature of the self-focusing effect. Based on this pattern, the corresponding value of the nonlinear refractive index for Ni/Cu/Ag TMNPs is calculated to be 18.40 × 10^–9^ cm^2^W^–1^. The origin of the nonlinear refractive index can be explained based on the thermal lens model. According to this model, in this case, nonuniform refractive index distribution acts like a converging lens as the CW laser beam passes through the medium.^72,73^

The third-order nonlinear susceptibility (χ^3^) indicates the effectiveness of the nonlinear response concerning the applied field. The magnitude of the susceptibility is computed by using both the real and imaginary components of the susceptibility.
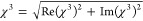
12

The optical nonlinearity of Ni/Cu/Ag TMNPs is attributed to various processes such as increased thermo-optical effects, orientation of polarizable groups, dipole moment, dielectric confinement, electronic excitation, and electrostriction.^74,75^ In CW laser Z-scan experiments, the thermo-optical effects are dominated by optical nonlinearity over other processes.^76,77^ The thermo-optical nonlinearity can be detected by measuring the samples’ peak–valley separation (⟨ΔZp–v⟩) of CA Z-scan curves. The peak–valley separation in the CA Z-scan curves of Ni/Cu/Ag TMNPs is measured, and the values are tabulated in Table 2. The table shows that the peak–valley separation is more than 1.7 times the Rayleigh range (Zo) for Ni/Cu/Ag TMNPs. This suggests that the optical nonlinearity originated from the thermo-optical effects.^78,79^ The thermo-optical impact arises from the spatial temperature distribution in the NLO materials.^80^ The spatial temperature distribution alters the material density and polarization within the materials. Therefore, the NLO properties of the materials may get altered drastically.^81^

**Table 2:** NLO Parameters of Ni/Cu/Ag TMNPs.

β×10^–5^ (cmW^–1^)	4.12
n_2_×10^–9^ (cm^2^W^–1^)	18.40
Re χ^(3)^× 10^–7^ (e.s.u)	24.60
Im χ^(3)^× 10^–8^ (e.s.u)	9.95
χ^(3)^× 10^–7^ (e.s.u)	24.60
OL (kJ/cm^2^)	3.2
*W*	18.944
*T*	0.011
ΔZp–v (cm)	5.57

**Figure 7:**
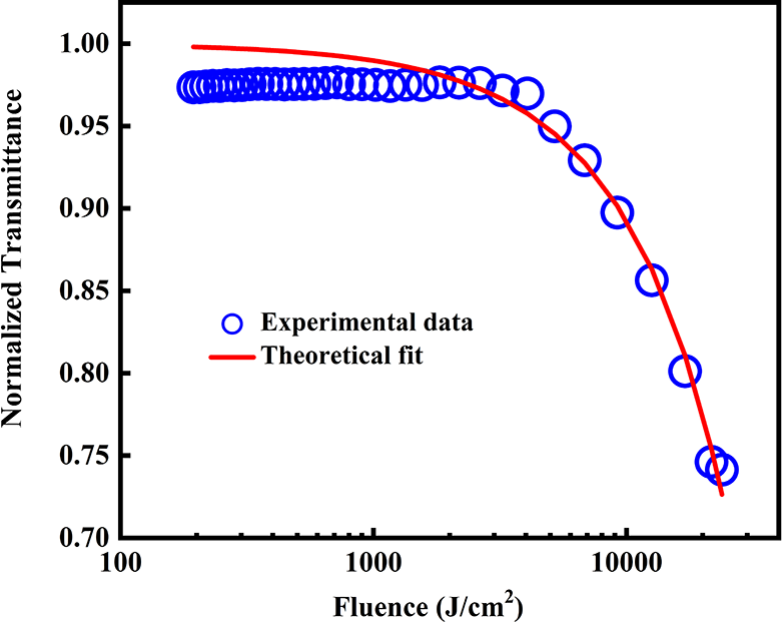
Optical limiting behavior of Ni/Cu/Ag TMNPs.

The optical limiting behavior of a material can be explained by various mechanisms, such as induced scattering, nonlinear refraction, free-carrier absorption, two-photon absorption, and reverse saturable absorption. In this study, the RSA behavior observed in TMNPs is attributed to thermally induced excited-state absorption, which leads to an increased absorption rate and decreased transmission at elevated laser fluences. Additionally, the self-focusing effect concentrates the laser beam, diminishing the transmission of the intense laser beams. Moreover, the surface LSPR effect and interband transitions significantly influence the optical limiting properties of metallic NSs.^82,83^ Optical limiting thresholds (OL) can be obtained by plotting the normalized transmittance versus the input fluence using the OA data to investigate the optical limiting performance of the samples (Figure 7). High transmittance for weak signals and opacity for significant optical signals are essential characteristics of a sensor or an optical device, as stated in.^84^ The input fluence at which the transmittance drops to half of the linear transmittance is known as the optical limiting threshold, a vital parameter to verify the feasibility of the material for optical switching and optical limiting applications.^85^ A decrease in the limiting threshold enhances the optical limiting performance of the material. In this study, the OL value of the Ni/Cu/Ag TMNPs was determined to be 3.2 kJ/cm^2^. The resulting TMNPs exhibited a lower limiting threshold, which is low compared to most of the previously reported metallic nanomaterial-based optical limiters. This makes them a highly desirable choice for optical sensors and devices exposed to intense laser beams. In addition, the figures of merit for one-photon (W) and two-photon (T) were more significant and less than unity, respectively, indicating that the prepared samples may also be suitable for optical switching devices.^65^ The comparison table (Table S1) visibly confirms that the in-depth study of NLO properties of metallic NSs under similar experimental conditions is sparse. Further, there are no reports on an investigation of the NLO behavior of TMNP NSs. Hence, the present study is novel and needs further exploration by optimizing the preparation and Z-scan parameters.

## 3 Conclusions

In conclusion, this study deals with the polyol synthesis of Ni/Cu/Ag TMNPs in ethylene glycol and explores their NLO properties. Morphological analysis, absorption spectra, XRD, and XPS confirmed the presence of an alloy structure in the TMNPs. OA Z-scan analysis revealed the RSA behavior, indicating strong NLO absorption. The calculated nonlinear absorption coefficient (β) confirmed that the prepared TMNPs exhibit thermally induced excited-state absorption. CA Z-scan analysis also demonstrated a self-focusing effect, indicating a nonlinear refractive index (n_2_). The characterization of Ni/Cu/Ag TMNPs regarding their NLO properties underscores their potential for optical limiting applications.

## 4 Experimental Method

### 4.1 Chemicals Used

Nickel (II) nitrate hexahydrate (H_12_N_2_NiO_12_, 98%), copper sulfate pentahydrate (CuSO_4_.5H_2_O, 99%), ethylene glycol, and silver nitrate (AgNO_3_, 99%) were procured from Loba Chemie Pvt. Ltd. Polyvinylpyrrolidone (87–90%) was purchased from Sigma-Aldrich. All of the chemicals were used without any further purification.

### 4.2 Method

Ni/Cu/Ag TMNPs were prepared using the polyol method, with ethylene glycol as the polyol and polyvinylpyrrolidone as the capping agent. The synthesis process involved the addition of 200 mg each of nickel (II) nitrate hexahydrate, copper (II) sulfate pentahydrate, and silver nitrate with 30 mL of ethylene glycol and 3 g of polyvinylpyrrolidone. The mixture was stirred for 60 min while maintaining a temperature of 180 °C, which caused changes in color until it reached a pale-green color, indicating the completion of the reaction. Once the reaction was finished, the solution was allowed to cool to room temperature. The schematic illustration of the synthesis process of the TMNPs is depicted in Figure S1 of the supplementary file.

### 4.3 Characterization

Optical studies of the prepared samples were carried out using a Shimadzu-1800 UV–vis spectrophotometer. Morphological and elemental analyses were conducted using a field emission scanning electron microscope and an energy-dispersive X-ray spectroscopy (FESEM; Carl Zeiss; EVO-18). TEM (Jeol ASIA PTE LTD; 2100 PLUS) was used to gain more insight into the morphology. Structural analysis was performed by XRD (Rigaku Miniflex 600). XPS (JEOL ASIA PTE LTD; JPS 9030) was used to conduct compositional analysis and determine the elements’ oxidation states. PL studies were carried out using a PL spectrometer (Jasco FP 8500).

### 4.4 Z-Scan Measurements

A continuous diode-pumped solid-state laser was used to obtain OA and CA Z-scan measurements at a wavelength of 532 nm and a 200 mW output power. The laser beam was focused by a plano-convex lens with a focal length of 28 cm, and the laser intensity was measured to be 1.81 × 10^8^ W/m^2^. The Rayleigh length and size of the beam waist for the given laser system are 3.98 × 10^–3^ and 2.59 × 10^–5^ cm, respectively. A glass cuvette with a path length of 1 mm holds the samples.

## Supporting Information Available

Schematic representation of the preparation of TMNPs and the obtained TMNPs (Figure S1), standard XRD pattern of monometallic counterparts (Figure S2), extinction spectra and variation of the refractive index with respect to wavelength (Figure S3), and comparison of NLO parameters of the present study with a few reported metallic NSs (Table S1) (PDF)
