# Supplementary material for: Polyol-Assisted Synthesis of Ni/Cu/Ag Trimetallic Nanoparticles for Nonlinear Optical Applications
Source: ACS Omega. 2024 Nov 15;9(47):46773–83. doi: 10.1021/acsomega.4c03143 (PMC11603202; doi:10.1021/acsomega.4c03143)
Supplement: Supplementary file 1 — ao4c03143_si_001.pdf [file ao4c03143_si_001.pdf]

Supporting information

Polyol-assisted synthesis of Ni/Cu/Ag trimetallic nanoparticles for non-linear optical applications

Shilpa Molakkalu Padre[a], Shivakumar Jagadish Shetty[a], Saideep Shirish Bhat[a], Desmond Gracian Rebello[a], Srivathsava Surabhi[b,c], Shreya Rao[a], Neelamma Basayya Gummagol[d], Maqsood Rafique Waikar[e], Rajendra Girjappa Sonkawade[e], Gurumurthy S C[a*]

[a]Nano and Functional Materials Lab (NFML), Department of Physics, Manipal Institute of Technology, Manipal Academy of Higher Education, Manipal -576104, Karnataka, India.

[b]Laboratorio de Nanocompuestos, Departamento de Ingeniería de Materiales (DIMAT), Facultad de Ingeniería (FI), Universidad de Concepción (UdeC), Concepción, Chile.

[c]Laboratorio de Nanociencias y Nanotecnología, Facultad de Ciencias Físico Matemáticas (FCFM), Universidad Autónoma de Nuevo León (UANL), San Nicolás de los Garza, Nuevo León, 66451, Mexico.

[d]Department of Physics, School of Advanced Sciences, KLE Technological University, Vidhyanagar, Hubballi-580031, Karnataka, India.

[e]Radiation and Materials Research Laboratory, Department of Physics, Shivaji University, Kolhapur-416004, Maharashtra, India.

Corresponding author: gurumurthy.sc@manipal.edu

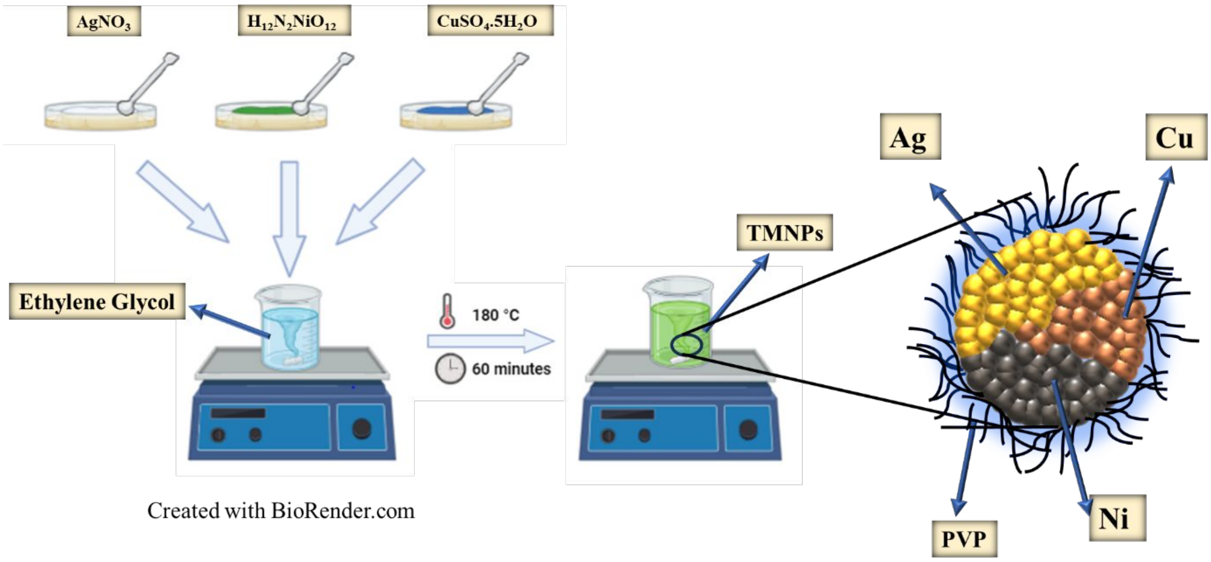

Figure S1. *Schematic illustration of the synthesis route and obtained of Ni/Cu/Ag TMNPs.*

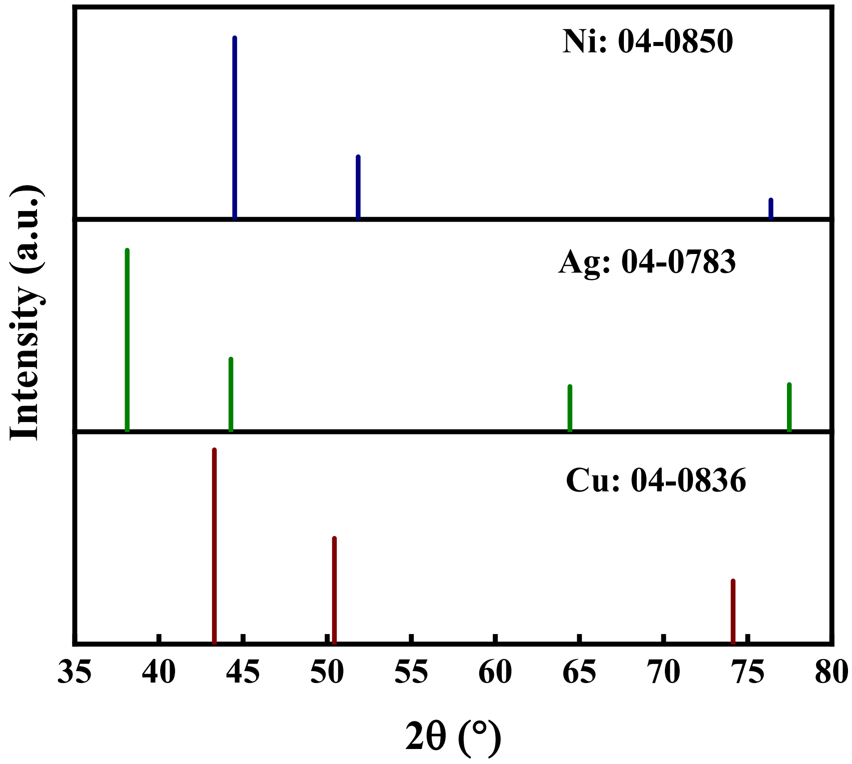

Figure S2. *Standard XRD pattern of Cu, Ag, and Ni monometals.*

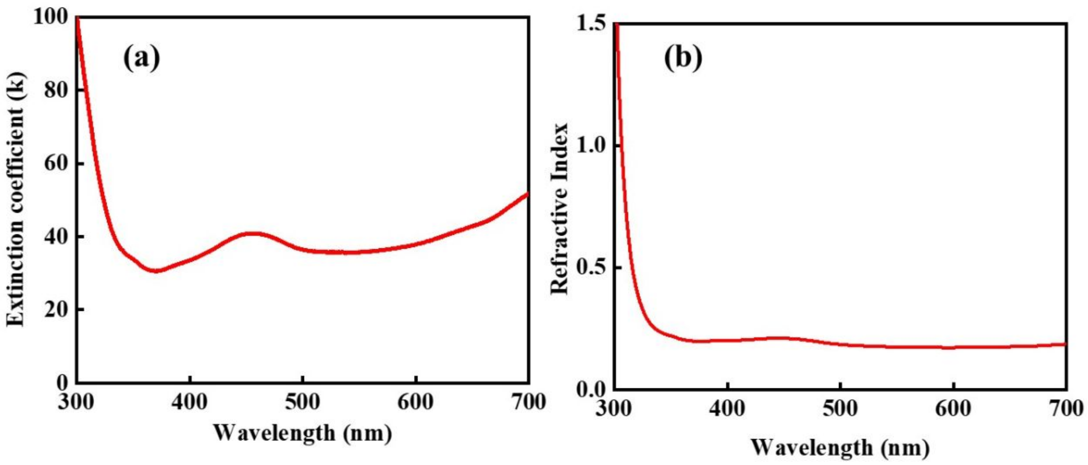

Figure S3. (a) Extinction spectra and (b) variation of refractive index with respect to wavelength of TMNPs.

Table S1. *Comparison of nonlinear optical parameters of prepared TMNPs with the few reported materials under similar experimental conditions.*

**Sample name**	**$\beta \times 10^{-4}$ (cmW^{-1})**	**$n_2 \times 10^{-8}$ (cm^2W^{-1})**	**$\chi^{(3)} \times 10^{-7}$ (e.s.u)**	**OL KJ/cm^2**	**References**
Au NPs	2	-1.7	-	-	[1]
Ag NPs	-	-5.04	-	-	[2]
Ag	-	-1	-	-	[3]
$CdFe_2O_4$-rGO	10	1	10	9.3–30.4 mW	[4]
Barium borate nanorod decorated on rGO	60.5	6.26	48.4	9.51 mW	[5]
PSAN/CdS polymer nanocomposite	5.61	0.29	1.71	-	[6]

P(Stco-MMA)/NiO polymer nanocomposite	3.82	1.83	10.50	-	[7]
Cadmium magnesium oxalate crystal	0.139	0.63	-	3.24	[8]
**Ni/Cu/Ag TMNPs**	**4.12**	**1.84**	**20.60**	**3.2**	**Present work**

References

(1) Azemoodeh Afshar, B.; Jafari, A.; Maqsood Golzan, M.; Naderali, R. Nonlinear Optical Properties of Gold Nanoparticles Produced by Laser Ablation at Two Different Radiation Wavelengths. *Results in Optics* **2023**, *12*, 100462. https://doi.org/10.1016/J.RIO.2023.100462.

(2) Faraji, N.; Yunus, W. M. M.; Kharazmi, A.; Saion, E. Third-Order Nonlinear Optical Properties of Silver Nanoparticles Mediated by Chitosan. *Optik (Stuttg)* **2014**, *125* (12), 2809–2812. https://doi.org/10.1016/J.IJLEO.2014.01.011.

(3) Karimzadeh, R.; Mansour, N. Thermo-Optic Nonlinear Response of Silver Nanoparticle Colloids under a Low Power Laser Irradiation at 532 Nm. *physica status solidi (b)* **2010**, *247* (2), 365–370. https://doi.org/10.1002/PSSB.200945377.

(4) Saravanan, M.; Sabari, S. G.; Vinitha, G. Facile Hydrothermal Synthesis of $CdFe_2O_4$-Reduced Graphene Oxide Nanocomposites and Their Third-Order Nonlinear Optical Properties under CW Excitation. *J Mol Liq* **2018**, *256*, 519–526. https://doi.org/10.1016/J.MOLLIQ.2018.02.065.

(5) Muruganandi, G.; Saravanan, M.; Vinitha, G.; Jessie Raj, M. B.; Sabari Girisun, T. C. Barium Borate Nanorod Decorated Reduced Graphene Oxide for Optical Power Limiting Applications. *Opt Mater (Amst)* **2018**, *75*, 612–618. https://doi.org/10.1016/J.OPTMAT.2017.11.017.

(6) Boranna, M. P.; Chandrakantha, K. S.; Gummagol, N. B.; Mahesh, S. S.; Patil, P. S.; Srikantaswamy, S.; Ravikumar, H. B. Studies on Microstructural Dependence of Nonlinear Optical Properties in PSAN/CdS Polymer Nanocomposites. *Journal of Materials Science: Materials in Electronics* **2023**, *34* (23), 1–15. https://doi.org/10.1007/S10854-023-11103-4/TABLES/4.

(7) Boranna, M. P.; Patil, P. S.; Gummagol, N. B.; Ravikumar, H. B. Positron Lifetime and Third-Order Nonlinear Optical Studies of P(St-Co-MMA)/NiO Polymer Nanocomposites. *Opt Mater (Amst)* **2023**, *140*, 113843. https://doi.org/10.1016/J.OPTMAT.2023.113843.

(8) Sanjeevannanavar, M. M.; Jagannatha, N.; Rohith, P. S.; Patil, P. S.; Gummagol, N. Third Order Non-Linear Optical Properties of Cadmium Magnesium Oxalate Crystals Grown by Silica Gel Technique. *Mater Today Proc* **2023**. https://doi.org/10.1016/J.MATPR.2023.03.604.
